# Zinc Finger 259 Gene Polymorphism rs964184 is Associated with Serum Triglyceride Levels and Metabolic Syndrome

**Published:** 2016

**Authors:** Seyed Reza Mirhafez, Amir Avan, Alireza Pasdar, Sara Khatamianfar, Leila Hosseinzadeh, Shiva Ganjali, Ali Movahedi, Maryam Pirhoushiaran, Valentina Gómez Mellado, Domenico Rosace, Anne van Krieken, Mahdi Nohtani, Gordon A. Ferns, Majid Ghayour-Mobarhan

**Affiliations:** 1 *Department of Basic Medical Sciences, Neyshabur University of Medical Sciences, Neyshabur, Iran.*; 2 *Department of Modern Science and Technologies; and Biochemistry of Nutrition Research Center; School of Medicine, Mashhad University of Medical Sciences, Mashhad, Iran.*; 3 *Division of Applied Medicine, Medical School, University of Aberdeen, Foresterhill, Aberdeen, AB25 2ZD, UK.*; 4 *Department of Medical Genetics, Faculty of Medicine, Mashhad University of Medical Sciences, Mashhad, Iran.*; 5 *VU University Medical Center, Amsterdam, De Boelelaan 1117, 1081 HV Amsterdam, The Netherlands.*; 6 *University of Bologna, Bologna, Italy.*; 7 *Peter MacCallum Centre, St Andrew's Place, Melbourne, Australia.*; 8 *Brighton & Sussex Medical School, Division of Medical Education, Falmer, Brighton, Sussex BN1 9PH, UK.*

**Keywords:** Metabolic syndrome, gene polymorphisms, lipid pathway

## Abstract

Metabolic syndrome (MetS) is characterized by a cluster of cardiovascular risk factors that include: abdominal obesity, dyslipidaemia, hypertension, insulin resistance and impaired glucose tolerance. Recent genome wide association studies have identified several susceptibility regions involved in lipid metabolism that are also associated with MetS. We have explored the association of 9 genetic polymorphisms involved in lipid metabolism and hypertension, including: *MTHFR* C677T, *SELE* L554F,* FGB *- 455G>A,* GNB3* C825T*, ZNF259 C>G, PSRC-1 *A>G*, CETP *I405V*, LPL *S447X and* LPA* C>T in 97 subjects with MetS and 96 individuals without MetS who were recruited randomly from Mashhad stroke and heart atherosclerotic disorder (MASHAD) study using a stratified cluster random sampling technique. Anthropometric parameters and biochemical measurements were determined in all the subjects. Genotyping was carried out followed by univariate and multivariate analyses. The subjects with MetS had a higher triglyceride and lower HDL- C. CG+ GG genotypes of *ZNF259 *polymorphism (rs964184 C>G) and TT+CT genotypes of *MTHFR* C677T (rs1801133) were associated with MetS, and individuals carrying the G allele for *ZNF259 *or the T allele for *MTHFR* polymorphisms were associated with MetS (e.g, odds ratio (OR) for CG+GG genotypes vs. CC wild type: 2.52, CI=1.33-4.77; P=0.005). However, after multiple comparison adjustment, this relationship remained significant only for CG+ GG genotypes of *ZNF259 *polymorphism. Moreover, the *ZNF259* CG+ GG genotypes were associated with increased serum concentrations of triglycerides and LDL-C, compared to the wild type. These data support the necessity for further studies in larger multicenter settings.

Metabolic syndrome (MetS) is a common condition comprising a cluster of cardiovas-cular risk factors including: abdominal obesity, dyslipidemia, hypertension, insulin resistance and impaired glucose tolerance. It is associated with an increased risk of cardiovascular disease (CVD) and diabetes mellitus (1). Over the past decades, the number of people with MetS has increased globally. The prevalence of MetS in the Iranian adult population (95% confidence interval) is approxim-ately 32.1% (31.2-33.0) by the International Diabetes Federation (IDF) definition, 33.2% (32.3-34.1) by the adult treatment panel III report  (ATPIII) and 18.4% (17.6-19.2) according to the WHO definition (2).

Genome wide association studies (GWAS) have identified several susceptibility loci and genes related to MetS and CVD; these include: Lipoprotein(a) (*LPA*)*, *lipoprotein lipase (*LPL*) and Cholesteryl ester transfer protein (*CETP*) (3, 4). Kraja et al. identified 29 unique variants in or near 15 genes. These genes were mainly related to lipid metabolism pathway, which has been shown to have an important role in the genetic background of MetS. Consistent with these observations, several other studies had shown that the most important variants in the correlation among traits of MetS were in or near *LPL*, *CETP* and *ZNF259* genes, which are known to play a key role in lipid metabolism (4-8). Lipoprotein (a) consists of a cholesterol-laden low-density lipoprotein (LDL)–like particle bound to a plasminogen-like glycoprotein, apolipoprotein (a). Lipoprotein (a) has been shown to be associated with thrombosis and atherosclerosis, and genetic data support a role for lipoprotein (a) in atherosclerotic stenosis and MetS (9-11).

Lipoprotein lipase (LPL) plays an important role in lipid metabolism and is expressed in the myocardium, adipose tissue and skeletal muscle (12). It catalyzes the hydrolysis of triacylglycerol present in chylomicron particles and VLDL (13). This reaction provides free fatty acids and mono-acylglycerol for use by skeletal, cardiac muscle and adipose tissue. The LPL gene is located on the short (p) arm of chromosome 8 at position 22. A common variant S447X (rs328) was reported at carrier frequencies of approximately 10 to 25% or higher frequencies in some populations (14-15).

CETP has been reported to have an association with CVD and its exact role in disease pathogenesis is unclear. CETP plays a key role in cholesteryl ester transfer from HDL-C to TG-rich lipoproteins. *CETP* polymorphisms are also associated with MetS (16) and increased level of TG and lower HDL- C levels (3). The CETP gene is located on the long (q) arm of chromosome 16 at position 21. It has been shown previously that polymorphisms in the *CETP* gene are related to increased risk for CAD (17-18).

High plasma concentration of homocysteine may predispose individuals to atherosclerosis by injuring the vascular endothelium, which might result in hypertension (19-21). The *MTHFR* gene is located on the short (p) arm of chromosome 1 at position 36.3. Another candidate gene, which might be involved with hypertension, is fibrinogen. Fibrinogen is a soluble glycoprotein, which is synthesized in the hepatocyte. Plasma fibrinogen is a dimer composed of three polypeptide chains, α, β and γ that are coded by three genes *FGA*, *FGB* and *FGG,* respectively (22, 23). It has been shown that plasma fibrinogen levels are associated with CVD, however, the role of genetic variation in the etiology of MetS still remains conflicting (22, 23). The *FGB* gene is located on the long (q) arm of chromosome 4 at position 28. The rs1800790 (-455 G/A) polymorphism within the promoter of *FGB* gene has been shown to be related with increasing plasma fibrinogen concentration (22, 24). Further-more, circulating markers of systemic inflammation such as SELE were shown to predict an increased risk of CVD. Endothelial leukocyte adhesion molecule-1 (selectin E) is a cell adhesion molecule that mediates the interaction of circulating leukocytes with vascular endothelium in various pathological and physiological settings (26). Leukocyte- endothelial interactions contribute to a variety of vascular disease processes such as atherosclerosis and chronic inflammation in metabolic disease. The *SELE* gene is located on the long (q) arm of chromosome 1 at position 24.2. Several studies have been reported that the L554F allele (rs5355) is associated with a higher risk of developing atherosclerosis and increased blood pressure risk in overweight individuals (27-28). In 1998, a common single nucleotide polymorphism (SNP), rs5443 (C825T) (thymidine to cytosine change), located on exon 10 of the *GNB3* gene was identified to be associated with CVD (29, 30). Several studies have shown that the T allele of the *GNB3* rs5443 SNP is associated with a number of health outcomes and other features of MetS including obesity, insulin resistance, dyslipidemia, hypertension (29, 30). SORT1 is involved in the uptake of LDL by the liver. Several studies have illustrated a significant positive correlation between PSRC1 and serum LDL concentrations (31-33). The risk allele of this variant on chromosome 11q23.3 (ZNF259, APOA5-A4-C3-A1 gene region) was associated with increased LDL cholesterol and decreased HDL cholesterol (and previously, with increased triglycerides) (34).

In the present study, we investigated the association of nine polymorphisms involved in lipid metabolism and hypertension, including *MTHFR* rs1801133, *SELE *rs5355,* FGB *rs1800790,* GNB3 *rs5443*, PSRC1 *rs599839*, LPL *rs328*, Lp(a) *rs3798220*, ZNF259 *rs964184* and CETP *rs5882 in

193 subjects with and without MetS.

## Materials and Methods

**Phenotypic definition of metabolic syndrome**

We used the IDF criteria to define MetS. Accordingly, a person with MetS would have central obesity (waist circumference (WC) in males ≥94 cm and in females≥80 cm) plus any two of the following four factors: TG level≥1.7 mmol/L (150 mg/dL) or specific treatment for this lipid abnor-mality; HDL-cholesterol< 1.03 mmol/L (40 mg/dL) in males and <1.29 mmol/L (50 mg/dL) in females or specific treatment for this lipid abnormality; systolic blood pressure (SBP)≥130 or diastolic blood pressure (DBP)≥85 mmHg or treatment of previously diagnosed hypertension; fasting plasma glucose (FPG)≥ 5.6 mmol/L (100 mg/dl) or previously diagnosed type 2 diabetes) (35).

**Population**

In the current study, 193 individuals, including 97 patients with MetS, and 96 healthy controls were recruited randomly from Mashhad stroke and heart atherosclerotic disorder (MASHAD) study, who were drawn from three regions in Mashhad, located in the north-eastern Iran, using a stratified cluster random sampling technique (36). Participants had no family history of stroke, myocardial infarction, and diabetes mellitus. Informed consent was obtained from all participants using study protocol approved by the Ethics Committee of Mashhad University of Medical Sciences.

**Anthropometric and Biochemical Measurements**

Anthropometric parameters (height, body weight, waist and hip circumference) were measured as described previously (36, 37). Body mass index (BMI) was calculated as body weight (kg) divided by squared height in meters (m^2^), and BMIs of 20–25, 25–30 or >30 were considered as normal, over-weight or obese, respectively. SBP and DBP were measured in duplicate by sphygmomanometer. Serum total cholesterol (TC), high density lipoprotein (HDL), low density lipoprotein(LDL) and triglyceride (TG), and fasting blood glucose (FBG) concentrations were evaluated by standard enzymatic techniques, while serum C-reactive protein(CRP) levels were determined by polyethylene glycol-enhanced immunoturbidimetry, as menticned beforehand (37).

**Genotyping**

Genomic DNA was extracted from peripheral blood leukocytes using QIAamp® DNA Mini-Kit (Qiagen, San Diego, CA) according to the manufacturer’s protocol at the VU university medical center Amsterdam. The concentration and purity of DNA samples was measured with the NanoDrop®-1000-Detector (NanoDrop-Technolo-gies, Wilmington, USA). Genotype analysis of *GNB3*, *FGB*, *MTHFR*, *SELE, PSRC1, LPL, Lp(a), ZNF259 *and* CETP *polymorphisms was performed using Taqman®-probes-based assay; PCR reactions were carried out in 12.5 µl total volume, using 20 ng of DNA in TaqMan® universal master mix with specific primers and probes (C-2184734-10, C-7429790-20, C-1801133-10, C-11975323-20, C-972962-10, C-901792-10, C-25930271-10, C-890762-10, C-790057-10; Applied Biosystems Foster City, CA). The ABIPRISM-7500 instrument equipped with the SDS version-2.0 software was utilized to determine the allelic content of the samples.

**Statistical analysis**

Data were analyzed using SPSS-20 software (SPSS Inc., IL, USA). The normality of distribution was determined using the Kolmogorov-Smirnov test. Descriptive statistics including mean, frequency, and standard deviation (SD) were determined for all variables and were expressed as mean± SD for normally distributed variables (or as the median and IQR for not normally distributed variables). For normally distributed variables, the student's t-test was used to compare the clinical characteristics and baseline demographics between the groups. A Bonferonni correction was applied for multiple comparisons. The Mann-Whitney U test was used for continuous variables if they were not normally distributed. Logistic regression analysis was used to calculate association of polymorphisms and MetS in the presence of confounders such as age, sex and smoking. All analyses were two- sided and statistical significance was set at p< 0.05.

## Results

**Characteristics of the population**

The baseline characteristics of the individuals with and without MetS are summarized in Table 1. Not surprisingly, subjects with MetS had a significantly higher triglyceride (TG), WC, SBP, smoking, HDL cholesterol (HDL-C); p< 0.05), while no differences were found for age, gender, BMI, weight, height, hip circumference (HC), serum TC, LDL- C, high-sensitivity CRP (HsCRP), DBP, FBG between the groups (Table 1).

**Polymorphisms and risk of MetS**

To investigate whether there was an association between *MTHFR* C677T, *SELE* L554F,* FGB*-455G>A,* GNB3* C825T, *ZNF259 C>G, PSRC1 A>G, CETP I405V, LPL S447X *and* LPA C>T *polymorphisms and MetS, we carried out genotyping using genomic DNA extracted from peripheral blood samples. Genotyping was success-fully performed in the vast majority of DNA samples, and no discrepancies were found in the samples analyzed in duplicate (approximately less than 10%).

As shown in Table 2, the wild- type *MTHFR*-rs1801133 genotype (CC) had a frequency of 49%, whereas the CT and TT genotypes were found in 43.8% and 7.3% of the control group, respectively, while these frequencies in the MetS group were 40.4% (CC), 41.5% (CT), 18.1% (TT). Moreover, individuals with the MTHFR- rs1801133-TT genotype or those who carried the T allele of the *MTHFR*-rs1801133 polymorphism were more likely to have MetS (p< 0.05, respectively).

Furthermore, 5.2% and 35.1% of MetS patients had the *ZNF259 *GG or CG genotype respectively, whilst CC genotype was found in 59.8% of the patients. However, these frequencies in the control group were 3.2%, 17.7 and 78.9 for GG, CG and CC genotypes (Table 2). The G allele of *ZNF259* variant increased the risk of MetS (OR=2.58, 95%CI=1.31-5.08; P=0.006). These findings were tested based on the ORs and their 95% CI for the association of the SNP with MetS using logistic regression. Furthermore, no significant differences were identified between the *MTHFR* and other polymorphic genotypes and groups after adjustment for age, sex, and smoking status (Table 2). All polymorphisms were consistent with the Hardy–Weinberg equilibrium, as calculated using the SNP analyzer software (http://snp.istech21.com/snpanalyzer/2.0/ ; Table 2) and their allelic frequencies were comparable to the reported population in the NCBI and NCI-SNP500 databases.

**Fig. 1:**
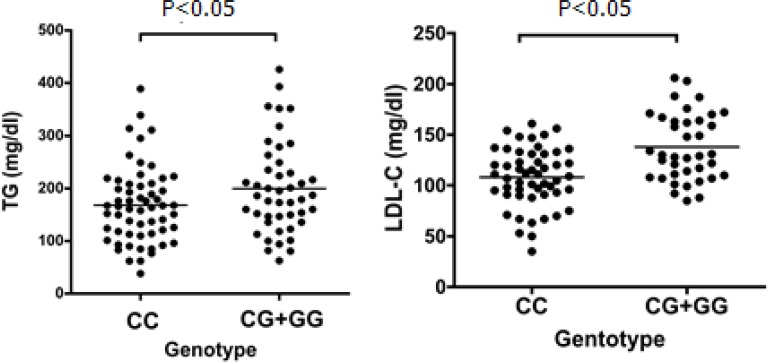
Association of *ZNF259* polymorphism with TG and LDL-C. Association of **(A)** TG and (B) LDL-C in MetS subjects with *T*-CG+GG genotypes versus control group

Additionally, we evaluated the association of the emerging genotypes with the components of MetS including WC, blood pressure (BP), HDL-C, FPG and TG levels. These analyses showed that CG+GG genotypes of *ZNF259 C>G *polymorphism were associated with an increased serum of LDL- C and TG, compared to the CC wild-type genotype (Figure 1). Other analyses related to MetS components such as WC, HDL - C, FPG and BP were not significant (data not shown). Additionally, we evaluated the association of the emerging genotypes with the components of MetS including WC, blood pressure (BP), HDL-C, FPG and TG levels. These analyses showed that CG+GG genotypes of *ZNF259 C>G *polymorphism were associated with an increased serum of LDL-C and TG, compared to the CC wild-type genotype (Figure 1). Other analyses related to MetS components such as WC, HDL-C, FPG and BP were not significant (data not shown).

## Discussion

This is the first study evaluating the association of *MTHFR*- rs1801133, *SELE*-rs5355,* FGB*-rs1800790,* GNB3*- rs5443*, PSRC1- *rs599839*, LPL- *rs328*, Lp(a)- *rs3798220*, ZNF259-*rs964184* and CETP-*rs5882 polymorphisms and MetS in Iranian patients. We demonstrated that GG genotype of *ZNF259 *was markedly associated with increased risk of MetS in our population. Moreover, consistent with several studies, no statistically significant association was detected for other SNPs with MetS (20, 25, 38- 40).

**Table 1.** Baseline characteristics of the individuals with and without metabolic syndrome

**Characteristics**	**Without MetS** **(n=96)**	**With MetS** **(n=97)**	**P value**
Age (y)	50.1±10.5	51.3±9.6	0.412
Gender, N(%) Male	31(32.3)	37(38.1)	0.395
Non- smokers, N (%)	13(13.5)	66(68)	<0.001
Weight (Kg)	71.9±11.1	75.1±11.9	0.062
Height (cm)	160±10	160±0.9	0.876
BMI (Kg/m^2^)	28.3±4.3	29.4±4.0	0.082
WC (cm)	91.8±11.2	98.6±8.4	<0.001
HC (cm)	102.1±7.7	105.0±8.2	0.132
SBP (mmHg)	124.2±20.8	130.4±19.8	0.036
DBP (mmHg)	81.6±11.819	84.5±12.3	0.103
TC (mg/dl)	199.2±40.9	165.9±37.2	0.563
LDL (mg/dl)	122.9±39.5	120.4±33.9	0.649
hsCRP (mg/dl)	1.8(1.1-3.5)	1.54(1.1-3.0)	0.584
HDL (mg/dl)	43.5±9.1	38.0±8.6	<0.001
FBG (mg/dl)	88.7±30.1	89.6±24.9	0.817
TG (mg/dl)	128.0(99.5-171.0)	177.5(135.0-230.5)	<0.001

Values are expressed as mean ±SD, median and interquartile range for normally and non-normally distributed variables, respectively. BMI: body mass index; WC: waist circumference, TC: total cholesterol; TG: triglycerides; HDL-C: high-density lipoprotein cholesterol; LDL-C: low-density lipoprotein cholesterol; FBG: fasting blood glucose; HC: hip circumference, SBP: systolic blood pressure; DBP: diastolic blood pressure; high-sensitivity C-reactive protein: HsCRP; Metabolic syndrome: MetS

**Table 2:** Crude associations between genotype, alleles and metabolic syndrome

	**Control**	**MetS**	**Odds ratio (95%CI)**	**P value**	* **Odds ratio (95%CI)**	* **P value**
**Rs5355 ** ***SELE***	96	97				
CC	77(80.2)	84(86.6)	Ref Cat			
CT	19(19.8)	13(13.4)	0.62(0.29-1.35)	0.235	0.37(0.13-0.99)	0.058
C	173(90)	181(93)	Ref Cat			
T	19(10)	13(7)	0.66(0.24-1.83)	0.435		
**rs1800790 ** ***FGB***	93	97				
GG	58(62.4)	50(51.5)	Ref Cat			
GA	27(29)	41(42.3)	1.76(0.95-3.26)	0.071	1.57(0.43-5.70)	0.493
AA	8(8.6)	6(6.2)	0.87(0.28-2.67)	0.808	0.51(0.25-1.04)	0.064
G	143(77)	141(73)	Ref Cat			
A	43(23)	53(27)	1.25(0.78-1.98)	0.347		
**rs5443 ** ***GNB3***	89	84				
CC	55(61.8)	44(52.4)	Ref Cat			
CT	25(28.1)	29(34.5)	1.45(0.74-2.82)	0.274	0.88(0.26-2.97)	0.849
TT	9(10.1)	11(13.1)	1.52(0.58-4.01)	0.390	1.12(0.53-2.34)	0.762
C	135(76)	117(70)	Ref Cat			
T	43(24)	51(30)	1.37(0.69-2.70)	0.360		
**rs1801133 ** ***MTHFR***	96	94				
CC	47(49)	38(40.4)	Ref Cat			
CT	42(43.8)	39(41.5)	1.14(0.62-2.11)	0.657	0.74(0.35-1.58)	0.446
TT	7(7.3)	17(18.1)	3.00(1.12-7.99)	0.028	2.06(0.63-6.69)	0.229
TT+CT	89(92.7)	77(81.9)	2.80(1.10-7.12)	0.030	2.37(0.77-7.24)	0.129
C	136(71)	115(61)	Ref Cat			
T	56(29)	73(39)	1.54(1.00-2.36)	0.047		
**rs5882 ** ***CETP***	95	97				
GG	44(46.3)	41(42.3)	Ref Cat			
GA	40(42.1)	43(44.3)	1.15(0.63-2.11)	0.643	0.67(0.31-1.43)	0.303
AA	11(11.6)	13(13.4)	1.26(0.51-3.14)	0.608	0.88(0.29-2.65)	0.823
G	128(67)	125(64)	Ref Cat			
A	62(33)	69(36)	1.14(0.74-1.73)	0.544		
**rs328 ** ***LPL***	96	96				
CC	77(80.2)	81(84.4)	Ref Cat			
CG	17(17.7)	15(15.6)	0.83(0.39-1.79)	0.651	1.41(0.58-3.45)	0.443
GG	2(2.1)	0(0)	<0.001	0.999	<0.001	0.999
C	171(89)	177(92)	Ref Cat			
G	21(11)	15(08)	0.69(0.34-1.38)	0.296		
**rs599839 ** ***PSRC1***	96	96				
AA	78(81.2)	77(80.2)	Ref Cat			
AG	15(15.6)	18(18.8)	1.21(0.57-2.58)	0.612	0.84(0.33-2.15)	0.728
GG	3(3.1)	1(1)	0.33(0.03-3.31)	0.352	0.14(0.01-2.02)	0.153
A	(89)171	172(90)	Ref Cat			
G	21(11)	20(10)	0.94(0.49-1.81)	0.869		
**rs964184 ** ***ZNF259***	95	97				
CC	75(78.9)	58(59.8)	Ref Cat			
CG	17(17.9)	34(35.1)	2.15(0.49-9.39)	0.307	3.25(1.43-7.38)	0.005
GG	3(3.2)	5(5.2)	2.58(1.31-5.08)	0.006	1.42(0.23-8.62)	0.701
CG+GG	20(21.1)	39(40.2)	2.52(1.33-4.77)	0.005	2.92(1.34-6.36)	0.007
C	167(88)	150(77)	Ref Cat			
G	23(12)	44(23)	2.13(1.22-3.69)	0.007		
**rs3798220 ** ***LPA***	88	91				
TT	75(85.2)	80(87.9)	Ref Cat			
TC	13(14.8)	11(12.1)	0.79(0.33-1.87)	0.599	0.96(0.34-2.71)	0.965
T	163(93)	171(94)	Ref Cat			
C	13(7)	11(6)	0.80(0.35-1.85)	0.612		

Ref Cat: Reference category, CI: Confidence interval; Logistic regression analysis was used to calculate association of polymorphisms and metabolic syndrome.

* After correction for age, sex and smoking

MetS and its components, including hypertension, hyperlipidemia, obesity, insulin resistance or glucose intolerance, and fatty liver diseases, significantly contribute to the increased risk of cardiovascular diseases and diabetes (1). In recent years, genome-wide association studies (GWASs) have identified many candidate polymorphisms that may be associated with metabolic-related traits (3, 4). These studies revealed the association of several genetic polymorphisms in lipid metabolism pathway with MetS. In particular they identified *LPL*, *CETP* and *ZNF259* as the most significant influential variants related to the traits of MetS (4-8). In addition, another recent GWAS has identified *LPA* variants as the most significant factor related to the CVD (9). Furthermore, Clarke et al. demonstrated a significant association between *LPA* variants with both an increased level of lipoprotein (a) and an increased risk of coronary disease (9). Clee et al. showed that S447X variant carriers had a trend toward decreased vascular disease, decreased triglyerides and decreased DBP compared to non-carriers (15). Previous studies reported that *LPL* variants are associated with individual components of MetS (5-7), as well as with insulin resistance and CVD (6, 7). In a case-control study of Ashkenazi Jewish families with exceptional longevity, an increased frequency in homozygosity for the V405 allele (rs5882) (VV genotype) was found. Long-lived individuals had lower serum CETP concentrations and increased lipoprotein sizes. They suggested that the inherited large lipoprotein particle sizes promoted a healthy aging phenotype (18). Wang et al. established a GWAS study in a case-control cohort and detected the association between myocardial infarction and four SNPs, including rs599839 near *PSRC1* and sortilin 1 (*SORT1*) gene on the chromosomal region of 1p13.3 (31). Furthermore, an intra-genic variant, rs964184, near *ZNF259* is found to be related to serum TG level and hyperlipidemia (33). By contrast, several other studies could not replicate these findings (20, 25, 38). In particular, Yamada and collaborators evaluated the association of candidate gene polymorphisms, e.g., *GNB3* 1429C>T, related to lipid metabolism in 2417 Japanese subjects, including 1522 with MetS and 895 controls. This study failed to show any relationship between *GNB3* and MetS (38). Consistent with this data, we found that this polymorphism was not associated with the disease in our population. Similarly, a recent meta- analysis by Povel et al. could not demonstrate a correlation between the C825T polymorphism and MetS (pooled OR of 825T vs. C: 1.03, 95%CI 0.94–1.12) (41). Additionally, Albert et al. examined the relationship between candidate polymorphisms in the fibrinogen gene and its association with plasma levels in 565 white, 476 African-American, 277 Hispanic and 370 Asian women participating in the Women’s Genome Health Study. They illustrated that the -455 G/A polymorphism was significantly associated with baseline plasma levels of fibrinogen and with increased CVD risk (24). However, several other studies evaluating functional polymorphisms in the fibrinogen gene and relationship with CVD events have generally showed a weak or no relationship (25), which is in line with our observation.

A common genetic variant in *MTHFR* gene, rs1801133 (677 C>T), leads to the reduction in the activity of the MTHFR enzyme, and increased plasma total homocysteine (tHcy) levels. *MTHFR* 677C>T polymorphism is shown to modulate total tHcy and folate metabolism (19) and has been shown to be the most frequent genetic causes for mild hyperhomocysteinemia (19). More recently, Yang et al. evaluated the association of 23 SNPs located within 17 candidate genes in 2014 subjects with overweight and obesity, diabetes, metabolic phenotypes. This study showed that *MTHFR* had a strong association with hypertension with an odd ratio (OR) similar to the result of a previous meta-analysis among Asian population (20, 21). Accumulating evidence has shown the relation between* MTHFR* rs1801133 polymorp-hisms with reduced MTHFR enzyme activity, CVD risk (42) and hypertension (21). Our analysis showed a statistically significant association of *MTHFR* with MetS. We found that subjects with the TT genotype had approximately 3-fold higher OR for MetS compared to the wild-type *MTHFR*-rs1801133 genotype (CC). However adjustment for age, sex and other confounding factors did change the statistical significance and the magnitude of the association, indicating that the association may be dependent on these confounding factors. We also analyzed the relationship between rs1800790, rs5355, rs599839, rs328, rs3798220 and rs5882 polymorphisms and MetS, adjusting for multiple covariates. However, we did not detect a statistically significant association between these polymor-phisms and MetS, which might be possible because of the low statistical power due to a small sample size or small genetic effect sizes. On the other hand, several candidate gene studies had been investigated and showed inconsistent results (25, 38). This lack of correlation could be explained by several factors, including variations in the life style, diet, severity of the disease, small sample size, ethnic origin and/or medications. 

The findings of this stndy is in agreement with, several studies that also demonstrated inconsistent data (39-41).

To the best of our knowledge, this is the first study showing the association of *ZNF259 *with lipid profile and MetS. Our data illustrated that individuals carrying G allele for *ZNF259* were at an increased risk of having MetS with OR of 2.52 (95% CI= 1.33- 4.77; P= 0.005). The results of our stndy, are consistent with several recent studies that showed its association with hyperlipidaemia and CVD (43). Aung et al. explored the association of the *ZNF259* with hypercholesterolaemia and hypertriglyceridaemia. They showed that *ZNF259* rs964184 SNP was associated with serum lipid levels and the presence of hyperlipidaemia (43). Braun et al. reported an association between the *BUD13-ZNF259* and serum TG level (44). We also found that patients carrying G genotype had a significantly higher level of LDL-C and TG, compared to the CC wild-type.

The main limitation of this study is its case control study design and small sample size or small genetic effect sizes. Additionally, we cannot exclude the heterogeneity which might be present in Iranian population, future studies in this population, and using more carefully defined ethnic groups are needed.

In conclusion, we demonstrated a significant association of *ZNF259 C>G polymorphism *with MetS and showed that patients with GG genotype or those who carried the G allele had approximately 2.5- fold increased risk for developing MetS, further studies on evaluating the role of genetic markers in MetS are recommended.
